# Development and Characterization of Lecithin-based Self-assembling Mixed Polymeric Micellar (*sa*MPMs) Drug Delivery Systems for Curcumin

**DOI:** 10.1038/srep37122

**Published:** 2016-11-16

**Authors:** Ling-Chun Chen, Yin-Chen Chen, Chia-Yu Su, Wan-Ping Wong, Ming-Thau Sheu, Hsiu-O Ho

**Affiliations:** 1 School of Pharmacy, College of Pharmacy, Taipei Medical University, Taipei, Taiwan; 2 Clinical Research Center and Traditional Herbal Medicine Research Center, Taipei Medical University Hospital, Taipei, Taiwan.

## Abstract

Self-assembling mixed polymeric micelles (*sa*MPMs) were developed for overcoming major obstacles of poor bioavailability (BA) associated with curcumin delivery. Lecithin added was functioned to enlarge the hydrophobic core of MPMs providing greater solubilization capacity. Amphiphilic polymers (sodium deoxycholate [NaDOC], TPGS, CREMOPHOR, or a PLURONIC series) were examined for potentially self-assembling to form MPMs (*sa*MPMs) with the addition of lecithin. Particle size, size distribution, encapsulation efficacy (E.E.), and drug loading (D.L.) of the mixed micelles were optimally studied for their influences on the physical stability and release of encapsulated drugs. Overall, curcumin:lecithin:NaDOC and curcumin:lecithin:PLURONIC P123 in ratios of 2:1:5 and 5:2:20, respectively, were optimally obtained with a particle size of < 200 nm, an E.E. of >80%, and a D.L. of >10%. The formulated system efficiently stabilized curcumin in phosphate-buffered saline (PBS) at room temperature or 4 °C and in fetal bovine serum or PBS at 37 °C and delayed the *in vitro* curcumin release. *In vivo* results further demonstrated that the slow release of curcumin from micelles and prolonged duration increased the curcumin BA followed oral and intravenous administrations in rats. Thus, lecithin-based *sa*MPMs represent an effective curcumin delivery system, and enhancing BA of curcumin can enable its wide applications for treating human disorders.

Curcumin (1,7-bis(4-hydroxy-3-methoxyphenyl)-1,6-heptadiene-3,5-dione) is an active ingredient extracted from the root of *Curcuma longa* and has long been consumed as a dietary spice^1^. In China and India, curcumin has been used for centuries for treating various disorders because of its antiinflammatory, antioxidant, antiseptic, analgesic, wound healing, antispasmodic, anticoagulant, and antimicrobial properties. The most beneficial effect of curcumin is the suppression of the proliferation of various tumor cells; thus, it is a potential agent against cancer^2^,^3^,^4^. Moreover, animal models and human studies have displayed the safety of curcumin even at a high dose of 8 g/day^5^. However, its therapeutic applications are restricted because of its low aqueous solubility, rapid hydrolysis in alkaline media, light instability, and hydrophobic characteristics, which result in pharmacokinetic (PK) drawbacks such as low absorption, poor bioavailability (BA; only 1% in rats), extensive metabolism, and rapid elimination^6^,^7^,^8^.

Many methods have been developed for improving the oral BA of curcumin, including adjuvant therapy with piperine, which interferes with glucuronidation metabolism, and the use of other delivery vehicles^9^ such as liposomes^10^, phospholipid complexes^11^, and nanoparticles^12^,^13^,^14^. Mohanty and Sahoo^15^ reported that encapsulating curcumin in glycerol monooleate increased stability by protecting curcumin from hydrolysis and biotransformation. Furthermore, encapsulated curcumin was more effective than free curcumin against different cancer cell lines under an *in vitro* condition because of enhanced cellular uptake, which resulted in the induction of apoptosis and reduction of cell viability. In mice, nanoparticulate curcumin exhibited a higher BA and longer half-life than did free curcumin. Curcumin encapsulated in monomethoxy poly(ethylene glycol)-poly(3-caprolactone); MPEG-PCL) micelles maintained cytotoxicity to C-26 colon carcinoma cells *in vitro*. Curcumin encapsulation in MPEG-PCL micelles improved the half-life and area under the plasma concentration time–curve (AUC) of curcumin *in vivo*. Moreover, intravenous application of curcumin micelles inhibited the growth of C-26 colon carcinoma *in vivo* and induced a stronger anticancer effect than did free curcumin^16^. Furthermore, the maximum concentration (Cmax) achieved using a single oral dose of 410 mg of curcumin micelles (women, 3.7 μmol/L; men 2.6 μmol/L) was higher than that achieved using 8 g of free curcumin^4^.

For nanoparticle drug delivery systems, the solubility of curcumin encapsulated with amphiphilic polymers is enhanced because the hydrophobic core of micelles provides the space required for encapsulating poorly soluble therapeutic agents and is responsible for drug solubility and release patterns. Moreover, the approach increases stability by providing protection from the reticuloendothelial system (RES) and beneficially modifies PK and biodistribution behaviors, thus increasing the BA. A small micelle size can facilitate achieving a favorable biodistribution^17^,^18^,^19^,^20^; nanosized micelles enable the extravasation and accumulation of therapeutic agents in tumor sites, which constitutes a passive targeting approach that functions through the enhanced permeability and retention (EPR) effect^21^,^22^. Micelles have an additional advantage of being easily reproducible and preparable on a large scale^23^.

One limitation of traditional micelles encapsulated in an amphiphilic polymer is that the solubility is determined by only one polymer. To improve the solubility, additional amphiphilic polymers are used for forming a novel delivery system, namely mixed polymeric micelles (MPMs). MPMs increase the volume of the hydrophobic core of each micelle by incorporating extra hydrophobic materials, providing a larger solubilization space for hydrophobic drugs. MPMs retains all aforementioned advantages of traditional micelles while increasing the solubilization capacity for poorly soluble drugs. Krishnadas *et al*.^24^ prepared a paclitaxel micelle system by using 1,2-distearoyl-sn-glycero-3-phosphoethanolamine-N-methoxy(poly(ethylene glycol)-2000 (DSPE-PEG2K) alone or a mixture of DSPE-PEG2K and egg-phosphatidylcholine; they concluded that the mixture increases solubility more than DSPE-PEG2K alone does. Therefore, MPMs offer synergistic properties such as drug stability, loading efficiency, and therapeutic efficacy superior to those offered by traditional one-component micelles^25^.

Phospholipids play a major role in nanoparticle drug delivery. In addition to their solubilization property, they have numerous advantages for incorporation into a carrier system for enhancing therapeutic efficacy. Maiti *et al*.^11^ prepared curcumin nanoparticles by using hydrogenated soy phosphatidyl choline, a phospholipid derivative, at a molar ratio of 1:1. The serum concentration of curcumin obtained from the nanoparticles was higher (Cmax: 1.2 μg/mL) than that obtained from free curcumin (Cmax: 0.5 μg/mL), and the nanoparticles maintained an effective curcumin concentration for a longer period in rats. The BA of curcumin-loaded phospholipid nanoparticles was 2.5 times greater than that of free curcumin. These results are in concordance with those of an *in vivo* report revealing that curcumin-formulated PC has higher peak plasma levels and AUC values than does free curcumin^26^. Lecithin, a hydrophobic mixture of naturally occurring phospholipids, is widely applied in the food and pharmaceutical industries and is considered a safe and biocompatible excipient. Lecithin being a kind of phospholipids functions as crucial components of the cell membrane to maintain membrane fluidity and an absorption enhancer to facilitate drug absorption^25^. Therefore, lecithin-based formulations increase the BA. An intravenous injection of lecithin-based docetaxel nanoparticles enhanced the antitumor effect and was biocompatible^27^. Hu *et al*.^28^ reported that lecithin-based nanoparticles exhibited a sustained release profile, with approximately 80% of docetaxel released within 72 h, and had an oral BA higher than that of a docetaxel solution (8.75% versus 2.40%). Therefore, lecithin-based formulations improve the therapeutic efficacy of poor oral absorption drugs^29^. Curcumin-loaded lecithin liposomes exhibited 4-fold increased absorption (based on the AUC) compared with free curcumin in 9 healthy volunteers^30^.

To address the problem of low curcumin BA, micelle technology has emerged as a prominent solution. The present study involved developing and characterizing curcumin-loaded MPMs based on the same concept as previously reported^31^ of using lecithin for improving the curcumin solubility and BA. In addition to lecithin, an additional amphiphilic polymer, namely sodium deoxycholate (NaDOC), d-alpha tocopheryl polyethylene glycol succinate (TPGS), CREMOPHOR, or a PLURONIC series, was examined in this study for potentially self-assembling to form MPMs (*sa*MPMs). Figure 1(a) was used to exemplarily illustrate how thin film mixtures of lecithin, amphiphilic polymer, and API were self-assembled to form API-loaded *sa*MPMs. We developed micelles capable of self-assembling with a particle size of <200 nm, encapsulation efficiency (E.E.) of >80%, and drug loading (D.L.) of >10%. Furthermore, the physicochemical (morphological observation and *in vitro* drug release) and *in vivo* PK properties of the optimal formulation of curcumin-loaded lecithin-based self-assembling MPMs (*sa*MPMs) were characterized.

## Results

### Optimization of lecithin-based curcumin-loaded *sa*MPMs

**Figure 1:**
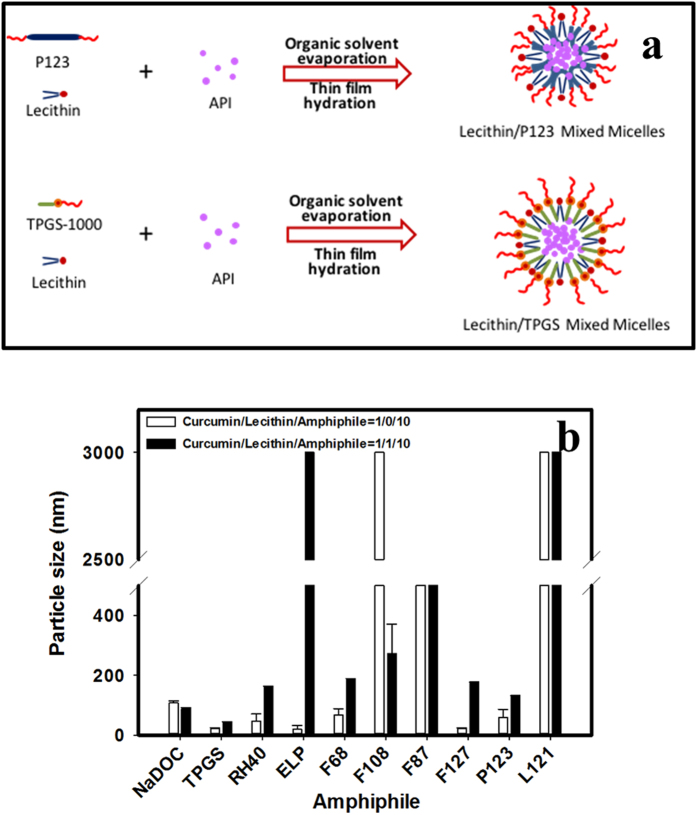
(**a**) Exemplified cartooning illustration of mixed micelles of lecithin/P123 and lecithicin/TPGS; (**b**) Particle size of curcumin micelles formed using different amphiphiles and ratios.

The particle size is a crucial parameter because it directly affects the physical stability, cellular uptake, biodistribution, and drug release from micelles, resulting in different BA values. The polydispersity index (P.I.) is a dimensionless measure of the breadth of the particle size distribution^32^,^33^. Tables 1 and 2 and Fig. 1(b) illustrate that the NaDOC micelles self-assembled with a particle size of <200 nm, and the addition of lecithin reduced the PI. The optimal formulation ratio for curcumin:lecithin:NaDOC was found to be 2:1:5. When a low TPGS concentration was used, the micelles precipitated within 12 h even in the presence of lecithin. As the concentration of TPGS was increased, the micelles were more stable, with particle sizes <100 nm. Regardless of the TPGS concentration, adding lecithin increased the E.E., but caused precipitation. When the CREMOPHOR concentration was low, the micelles precipitated within 12 h even in the presence of lecithin. Furthermore, with an increase in the CREMOPHOR concentration, the particle size decreased. However, adding lecithin increased the particle size and reduced the EE. The particle size of curcumin-loaded *sa*MPMs utilizing CREMOPHOR ELP was smaller than that of curcumin-loaded *sa*MPMs using CREMOPHOR RH40.

The particle size of curcumin-loaded *sa*MPMs utilizing PLURONIC flake series containing 70% EO fragments (i.e., F87 and F127) was smaller than that of curcumin-loaded *sa*MPMs using PLURONIC flake series containing 80% EO fragments (i.e., F68 and F108). Moreover, the F87 and F127 micelles were more stable and formed at a lower concentration; but adding lecithin increased the particle size. For PLURONIC liquid series, regardless of the ratio of PLURONIC L121 and lecithin, no transparent micelle solution was formed. When a lower PLURONIC P123 concentration was used, the micelles precipitated within 12 h even in the presence of lecithin. With an increase in the PLURONIC P123 concentration, the particle size decreased and stability increased. PLURONIC P123 facilitated micelle formation with a particle size of <200 nm, and adding lecithin increased the size, stability (i.e., no precipitation occurred at 12 h), and EE. The optimal ratio of curcumin:lecithin:PLURONIC P123 was found to be 5:2:20. Overall, NaDOC and PLURONIC P123 were the optimal polymers for lecithin-based curcumin-loaded MPMs, and the ratio of curcumin:lecithin:NaDOC was 2:1:5 and that of curcumin:lecithin:PLURONIC P123 was 5:2:20. The zeta potential for them was −39.6 ± 0.6 mV and −23.6 ± 0.15 mV, respectively. These two optimal formulations were selected for further evaluation.

**Figure 2:**
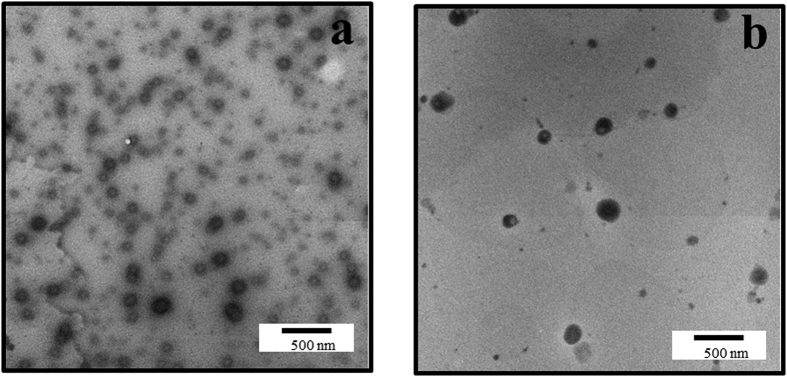
TEM micrograph of lecithin-based mixed curcumin micelles. (**a**) Curcumin:lecithin:NaDOC in a ratio of 2:1:5; (**b**) Curcumin:lecithin:PLURONIC P123 in a ratio of 5:2:20.

TEM revealed that the particle size was <200 nm for both optimal formulations (Fig. 2). The sizes of micelles formed by NaDOC were evener than those formed by PLURONIC P123, and the result was consistent with that of DLS, as shown by lower P.I. values.

### Characterizations of Optimal Formulations

**Figure 3:**
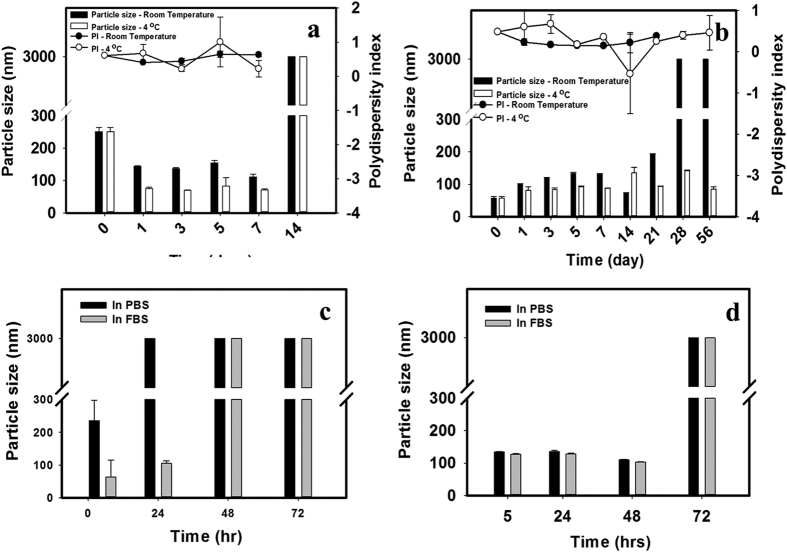
Stability of lecithin-based mixed curcumin micelles. (**a**) Curcumin:lecithin:NaDOC in a ratio of 2:1:5 in PBS at 4 °C or room temperature; (**b**) curcumin:lecithin:PLURONIC P123 in a ratio of 5:2:20 in PBS at 4 °C or room temperature; (**c**) Curcumin:lecithin:NaDOC in a ratio of 2/1/5 in PBS or FBS at 37 °C; (**d**) curcumin:lecithin:PLURONIC P123 in a ratio of 5:2:20 in PBS or FBS at 37 °C.

To confirm whether our optimal formulation increased curcumin stability, we incubated free curcumin and curcumin-loaded *sa*MPMs with PBS and FBS, respectively, and determined the time when the particle size was <200 nm, as shown in Fig. 3. In PBS, the particle size of curcumin-loaded NaDOC MPMs was >200 nm and was stable for <14 days at either room temperature or 4 °C (Fig. 3(a)). By contrast, curcumin-loaded PLURONIC P123 *sa*MPMs precipitated on Day 28 at room temperature, and at 4 °C, the size was not >200 nm even on Day 56 (Fig. 3(b)). At 37 °C in FBS, the particle size of curcumin-loaded NaDOC *sa*MPMs increased to >200 nm at 48 h (Fig. 3(c)). However, the size was not >200 nm until 72 h for curcumin-loaded PLURONIC^®^ P123 *sa*MPMs (Fig. 3d)).

**Figure 4:**
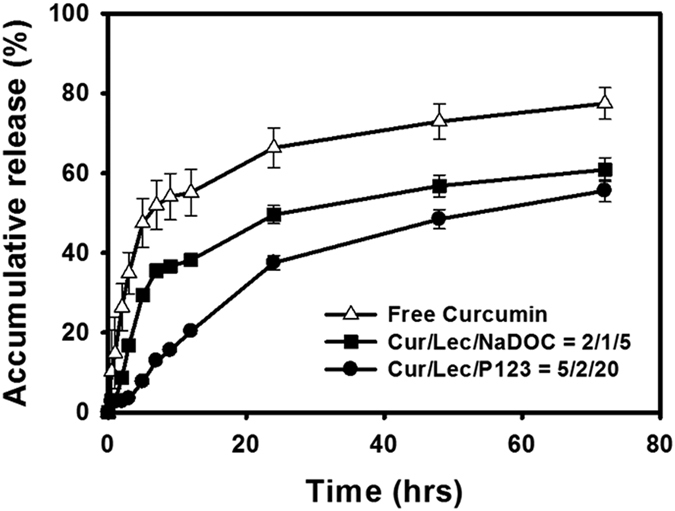
*In vitro* release profiles of curcumin from mixed micelles.

Figure 4 illustrates that the release rates of NaDOC and PLURONIC P123 MPMs were slower than that of the free curcumin solution. The release of curcumin-loaded PLURONIC P123 *sa*MPMs was slower than that of curcumin-loaded NaDOC *sa*MPMs.

**Figure 5:**
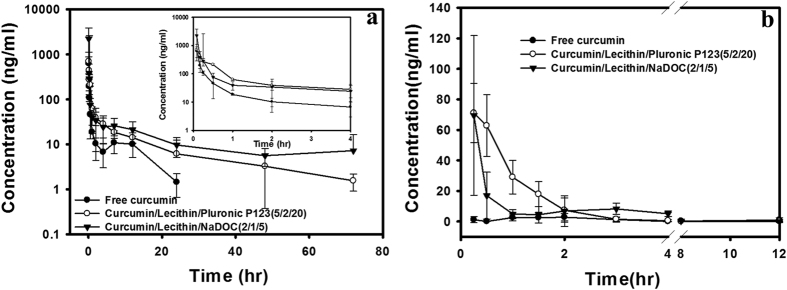
(**a**) Plasma concentration–time curves of curcumin after intravenous administration of lecithin-based mixed micelles (NaDOC and PLURONIC P123) and free curcumin (5 mg/kg) to rats; (**b**) Plasma concentration–time curves of curcumin after oral administration of lecithin-based mixed micelles (NaDOC and PLURONIC P123) and free curcumin (100 mg/kg) to rats. Each point represents the mean ± SD of 3 determinations (n = 3).

After the intravenous administration of free curcumin, the curcumin concentration quickly decreased and could not be detected after 24 h; however curcumin-loaded *sa*MPMs could be detected at 72 h (Fig. 5(a)). The MPMs reduced the elimination rate and increased the retention time of curcumin. The absolute BA was 232% and 573% for curcumin-loaded NaDOC *sa*MPMs and PLURONIC P123 *sa*MPMs, respectively (Table 3). The release rate and amount were higher for curcumin-loaded NaDOC *sa*MPMs than for curcumin-loaded PLURONIC P123 *sa*MPMs.

For oral administration, the maximal concentration of curcumin-loaded NaDOC and PLURONIC P123 *sa*MPMs was 15- and 17.5-fold higher than that of free curcumin, respectively (Fig. 5(b)). The absolute BA was 300% and 500% for curcumin-loaded NaDOC and PLURONIC P123 *sa*MPMs, respectively (Table 3). The release rate and amount were higher for curcumin-loaded NaDOC *sa*MPMs than for curcumin-loaded PLURONIC P123 *sa*MPMs.

## Discussion

The potential efficacy of curcumin in treating various diseases is a valuable research topic, and several clinical trials have determined its therapeutic effect. The pharmacological efficacy and safety make curcumin a prospective compound for treating and preventing various human diseases; however, curcumin has limited clinical applications because of its extremely low aqueous solubility, fast systemic elimination, insufficient tissue absorption, and degradation at an alkaline pH, severely curtailing the BA and limiting its clinical use. The maximum plasma concentration of curcumin in humans, even at a dose as high as 10 or 12 g, remains extremely low (<160 nmol/L)^34^. Furthermore, curcumin is classified as a biopharmaceutical classification system class IV molecule on the basis of its poor aqueous solubility (11 ng/mL in an aqueous buffer at pH 5) and low permeability through intestinal epithelial cells^3^,^35^,^36^. Therefore, the relative low solubility and BA of curcumin constitute a major problem, hindering its clinical use in exerting the maximum therapeutic activity against various diseases. To investigate the potential of using micelles as a drug delivery system for enhancing solubility and BA, the present study used amphiphilic polymers for developing curcumin micelles.

Lecithin, a natural mixture of phospholipids, is a major constituent of cell membranes, nervous tissues, and brain substances. Phospholipids possess a positively or negatively charged head group and a hydrocarbon tail, which has a zwitterionic head group at a physiological pH. Lecithin is a typical amphiphilic phospholipid with favorable biocompatibility and facilitates encapsulated drug absorption. However, the hydrophobic part alone is too short for forming a micelle. *sa*MPMs comprising 2 polymers can offer the benefits of both polymers, and the polymers can compensate for each other’s limitations; therefore, such MPMs are considered stable and were used in the present study.

The major factors affecting the particle size, D.L., and E.E. of *sa*MPMs are the polymer type and amount. In addition to lecithin, NaDOC, TPGS, CREMOPHOR RH40 and ELP, a PLURONIC flake series (F87, F127, F68, and F108), PLURONIC L121, or PLURONIC P123, was added for forming micelles. Micelles with PLURONIC F87 and F127 can be formed at a concentration lower than that of micelles with PLURONIC F68 and F108. Alexandridis^37^ reported that forming micelles with a higher EO fragment required a higher critical micelle concentration (CMC); therefore, a lower concentration was required for PLURONIC F87 and F127 for forming stable micelles. For the PLURONIC flake series, the ratio of hydrophilic PEO fragments was too high, and the PLURONIC surfactants were not compatible with the hydrophobic lecithin, causing curcumin-loaded PLURONIC F87, F127, F68, and F108 micelles to precipitate. Although the CMC of PLURONIC L121 is considered the lowest, and this surfactant can self-assemble into micelles, the PPO fragment is excessively long and has limited space for loading hydrophobic curcumin. Therefore, the micelles with PLURONIC L121 precipitated^38^,^39^,^40^,^41^. Combining TPGS and lecithin also caused precipitation. Moreover, using lecithin and CREMOPHOR increased the particle size and reduced the E.E.

Using NaDOC caused the self-assembly of small-particle micelles, the P.I. of which was lower than that of micelles formed using other amphiphilic polymers, indicating the even distribution. Adding PLURONIC P123 resulted in the formation of micelles with a particle size of <200 nm, and adding lecithin increased the particle size, stability, and E.E. The PEO fragment ratio of PLURONIC P123 is between that of PLURONIC L121 and the Flake series, and the ratio of hydrophobic to hydrophilic fragments of PLURONIC P123 is appropriate for forming stable micelles with lecithin and curcumin. Furthermore, a small particle size is beneficial for passive targeting to tumor tissues through the EPR effect, cellular uptake, and intracellular trafficking. Hence, we infer that the small size of the formulated curcumin micelles improved the circulation half-life and enabled avoiding the RES.

A major challenge in delivering curcumin to cancerous tissue is its instability and biodegradation at a physiological pH. Curcumin is stable in the stomach and small intestine but unstable in neutral and basic environments^42^,^43^. For studying curcumin biodegradation and stability, we incubated curcumin (free and curcumin-loaded *sa*MPMs) in PBS at room temperature, 37 °C, or 4 °C or in FBS at 37 °C and determined its particle size over time. In PBS and FBS, the micelle formulation increased curcumin stability by protecting the encapsulated curcumin against hydrolysis. Hence, the formulated system efficiently increased curcumin stability. Regardless whether the curcumin was incubated in FBS or PBS, the time when the particle size was <200 nm was longer for PLURONIC P123 than that for NaDOC. The CMC value of PLURONIC P123 was lower than that of NaDOC, revealing a higher stability of PLURONIC P123 in FBS. The lack of adherence of plasma proteins on the PLURONIC micelles was possibly due to the PEO units in PLURONIC, which can prevent protein adsorption. According to the stability test, curcumin-loaded *sa*MPMs utilizing PLURONIC P123 were more stable than those using NaDOC; therefore, the *in vitro* release rate of curcumin-loaded *sa*MPMs with PLURONIC P123 was slower.

Curcumin-loaded micelles are designed for improving the BA of the delivered curcumin. Therefore, curcumin-loaded *sa*MPMs and free curcumin were intravenously injected or orally administered in rats for monitoring the BA. Our results suggested the slow release of curcumin from micelles, reduced degradation, and prolonged duration increased the BA. However, for free curcumin, the concentration decreased with time and was not detected beyond 24 h and 2 h after oral and intravenous administration, respectively, indicating the rapid metabolism and excretion of free curcumin at a physiological pH. The degradation possibly resulted from rapid hydrolysis and biotransformation of curcumin into its glucuronide and sulfate conjugates within a short period. Moreover, for intravenous injection, unlike the observation for free curcumin, both curcumin-loaded PLURONIC P123 and curcumin-loaded NaDOC *sa*MPMs were detected for a long duration (after 24 h). For oral administration, the maximum plasma concentration of curcumin was observed after 0.33 h and 0.25 h for curcumin-loaded PLURONIC P123 and curcumin-loaded NaDOC *sa*MPMs, respectively; however, it was 1.75 h for free curcumin. The micelle biodistribution mainly depends on components of the hydrophilic shell causing the micelles to stabilize and interact with plasma proteins and cell membranes. Moreover, because of their amphiphilic characteristics, the encapsulated polymers used here have surfactant properties and offer stability and biocompatibility to micelles. For intravenous administration, the aforementioned surface-coated hydrophilic polymers are required for minimizing opsonization and for prolonging the *in vivo* micelle circulation. Irrespective of whether administration was intravenous or oral, curcumin-loaded PLURONIC P123 MPMs had a longer half-life and larger AUC than did curcumin-loaded NaDOC *sa*MPMs. The release rate and amount were higher for curcumin-loaded NaDOC *sa*MPMs than for curcumin-loaded PLURONIC P123 *sa*MPMs. *In vitro* release and stability study results revealed that curcumin-loaded PLURONIC P123 *sa*MPMs were more stable and released curcumin more slowly *in vitro*, indicating a greater BA.

Conclusively, we reported a simple and cost effective formulation composed of lecithin-based MPMs that was able to achieve the same degree of improvement in PK profiles and BA of curcumin as those reported PLGA formulations which was an expensive material^13^,^14^,^44^,^45^. Most encouragingly, curcumin is classified as a biopharmaceutical classification system class IV molecule on the basis of its poor aqueous solubility (11 ng/mL in an aqueous buffer at pH 5) and low permeability through intestinal epithelial cells. The current *sa*MPMs system for curcumin demonstrated that when curcumin-loaded *sa*MPMs were orally administrated, the time to achieve maximum plasma concentration of curcumin (Tmax) was shortened from 1.75 h for free curcumin to 0.33 h and 0.25 h for curcumin-loaded PLURONIC P123 and curcumin-loaded NaDOC *sa*MPMs, respectively, the maximal plasma concentration (Cmax) of curcumin-loaded NaDOC and PLURONIC P123 *sa*MPMs was 15- and 17.5-fold higher than that of free curcumin, respectively, and the absolute BA was 300% and 500% for curcumin-loaded NaDOC and PLURONIC P123 *sa*MPMs, respectively. It indicates that *sa*MPMs is able to not only improve the solubility of curcumin but also enhance its permeability through intestinal epithelial cells resulting in a significantly shorter Tmax, higher Cmax and larger BA. Ultimately, the process to produce lecithin-based *sa*MPMs is also simple and easily reproducible and preparable on a large scale.

## Conclusion

Considering the potential of micelles as a drug delivery system, the present study involved developing and characterizing lecithin-based curcumin *sa*MPMs for improving the curcumin BA. The optimal formulations of curcumin:lecithin:NaDOC and curcumin:lecithin:PLURONIC P123 were 2:1:5 and 5:2:20, respectively, providing a particle size of <200 nm, an E.E. of >80%, and a D.L. of >10%. Compared with free curcumin, the formulated system efficiently improved curcumin stability in PBS at room temperature, 4 °C, and 37 °C and in FBS at 37 °C, and retarded *in vitro* curcumin release. *In vivo* PK studies revealed that for oral administration, the absolute BA increased 3- and 5-fold for curcumin-loaded NaDOC and PLURONIC P123 *sa*MPMs, respectively. For intravenous administration, the absolute BA increased 2.32- and 5.73-fold for curcumin-loaded NaDOC and PLURONIC P123 *sa*MPMs, respectively. Thus, the slow release of curcumin from micelles, reduced degradation, and prolonged duration possibly increased the BA. Lecithin-based *sa*MPMs improve the solubility, stability, and BA of curcumin, representing an efficient delivery system. Therefore, increasing the curcumin BA by using lecithin-based *sa*MPMs can make curcumin a prominent therapeutic agent for treating human disorders.

## Materials and Methods

### Materials

Curcumin (total curcuminoid content, 95%) extracted from the rhizome of turmeric was purchased from Alfa Aesar (MA, USA). L-α-lecithin granules were supplied by Acros (NJ, USA). PLURONIC F87, F127, and F68; TPGS; and CREMOPHOR (ELP and RH40) were purchased from BASF (Hanover, Germany). Furthermore, sodium deoxycholate (NaDOC), PLURONIC L121, F108, and P123 were purchased from Sigma (MO, USA). DSPE-PEG2K was obtained from NOF (Tokyo, Japan), and heparin (5000 IU/mL) was provided by China Chemical & Pharmaceutical (Hsinchu, Taiwan). All reagents for high-performance liquid chromatography (HPLC) or ultra-performance liquid chromatography (UPLC)/mass-spectrometry (MS)/MS analysis were of an HPLC or MS grade, and other reagents were of an analytical grade.

### Preparation of curcumin-loaded *sa*MPMs

Curcumin-loaded *sa*MPMs were prepared using a thin film method, as previously described^46^. Briefly, curcumin, lecithin, and another polymer (NaDOC; PLURONIC F87, F127, F68, L121, F108, or P123; TPGS; or CREMOPHOR RH40 or ELP) in a predetermined ratio were added to 1 mL of a mixed solvent (methanol:dichloromethane, 3:7, v/v) in a round bottom flask. The mixture was shaken for 30 s, sonicated for 1 min, and subsequently evaporated through rotary evaporation (Buchi, Rotavapor R124, Switzerland) under reduced pressure for removing the solvent and obtaining a thin film. The self-assembly of the thin films resulting in micelle formation was induced by adding 1 mL of deionized water and gently shaking the micelle solution until the thin film completely dispersed. The unincorporated curcumin aggregates were removed by passing the solution through a 0.22-μm filter (Millipore, MA, USA). The characteristics of the curcumin-loaded *sa*MPMs, namely the average particle size and size distribution, E.E., and D.L. were determined.

### Characterization of curcumin-loaded *sa*MPMs

The average diameter and size distribution (Polydispersity index, P.I.) of the curcumin-loaded *sa*MPMs dispersed in water were measured via dynamic light scattering (DLS) mechanism using an N5 submicron particle size analyzer (Beckman Coulter, Brea, CA, USA). The intensity autocorrelation of the samples was adjusted in the range of 5 × 10^4^–1 × 10^6^ and measured at a scattering angle of 90° at room temperature. The particle charge was quantified as zeta potential using a Nano ZS90 (Malvern, UK). For observation of their surface morphology by transmission electron microscopy (TEM), an aqueous dispersion of *sa*MPMs were allowed to adsorb onto a carbon-coated grid and the *sa*MPMs were negatively stained with 2% (w/v) uranyl acetate (UA) for 30 s. Grid was then dried at room temperature and then subjected to examine by TEM (Hitachi H-700, Hitachi Ltd., Tokyo, Japan).

#### Quantification of curcumin

Curcumin was analyzed using an HPLC method (Pump PU-980, Jasco, Tokyo, Japan) adapted from Yang *et al*.^47^. The curcumin concentration was determined using an XBridge C18 column (5 μm, 150 × 4.6 mm). The mobile phase was a mixture of methanol and 0.3% acetic acid (7:3, v/v) at a flow rate of 1.0 mL/min at 30 °C. Furthermore, the column effluent was monitored using an ultraviolet detector (UV-975, Jasco, Tokyo, Japan) at a wavelength of 425 nm, and the HPLC method was validated to have an acceptable coefficient of variation for accuracy and precision. On determining the curcumin concentration from the validated calibration curve, the E.E. and D.L. were calculated according to equations (1) and (2), respectively:


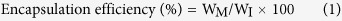



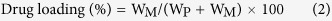


where W_M_ is the drug weight in micelles, W_I_ is the weight of the initial feeding drug, and W_P_ is the weight of the initial feeding polymers.

#### Stability test

The curcumin-loaded *sa*MPMs were stored at room temperature or 4 °C under dark conditions. At a predetermined time-point, the particle size of the curcumin-loaded *sa*MPMs was analyzed for evaluating the stability of their product. An equal volume of curcumin-loaded *sa*MPMs and phosphate-buffered saline (PBS; 0.01 M, pH 7.4) or fetal bovine serum (FBS) was co-incubated in a 37 °C water bath. At a predetermined time-point, the particle size of the curcumin-loaded *sa*MPMs was analyzed for evaluating their stability in plasma.

#### *In vitro* release studies

Drug release from the curcumin-loaded *sa*MPMs was assessed using the dialysis bag method, in which 0.01 M PBS containing 0.5% Tween 80 was used^48^. One milliliter of curcumin-loaded *sa*MPMs or a free curcumin solution (i.e., curcumin dissolved in dimethyl sulfoxide [DMSO]) diluted with water to yield a final concentration of 0.1 mg/mL was placed in a separate dialysis bag (MWCO 3500; Cellu-Sep T1, USA). The bag was placed in a tube, 20 mL of a dissolution medium was added, and the bag was placed at 37 °C at a shaking rate of 100 rpm. At 0.5, 1, 2, 3, 5, 7, 9, 12, 24, 48, and 72 h, concentration of curcumin released from the dialysis bag was analyzed using the HPLC method, as described in Section 2.4. All measurements were conducted in triplicate. For comparison, curcumin release from a free solution under the same conditions was assessed.

#### *In vivo* PK studies

This study involved an animal experiment that was approved by the Institutional Animal Care and Use Committee of Taipei Medical University (approval number: LAC-2013-0126) and conducted in compliance with the Animal Welfare Act. We used 8~10-week-old male Sprague-Dawley rats for investigating the PK profile of the optimal curcumin-loaded *sa*MPMs formulation (6 mg/mL) and a free curcumin solution (i.e., curcumin was dissolved in DMSO/PEG 400 at a ratio of 1:4 and then mixed with a 5% glucose solution to yield a final concentration of 6 mg/mL). Six rats were administered a single intravenous dose of 5 mg/kg of the curcumin-loaded *sa*MPMs or free curcumin solution (n = 3 for each group). Blood samples were collected in heparinized tubes from the jugular vein at 0.083, 0.166, 0.25, 0.5, 1, 2, 4, 7, 12, 24, 48, and 72 h after administration. In addition, 6 rats were orally administered a single dose of 100 mg/kg of the curcumin-loaded *sa*MPMs or free curcumin solution (n = 3 for each group). Blood samples were collected in heparinized tubes from the jugular vein at 0.25, 0.5, 1, 1.5, 2, 3, 4, 6, 8, 12, 24, 48, and 72 h after administration. All blood samples were immediately centrifuged at 3000 rpm for 15 min at 4 °C to obtain plasma, which was stored at −80 °C before UPLC/MS/MS analysis.

The UPLC/MS/MS analysis was performed according to a method adapted from Yang *et al*.^47^ by using the Waters ACQUITY UPLC and Xevo TQ MS system (Waters, Milford, MA, USA) equipped with an electrospray ionization (ESI) source. Separation was achieved using a BEH C_18_ column (2.1 mm I.D. × 50 mm, 1.7 μm; Waters, Milford, MA, USA). The system delivered a constant flow at 0.2 mL/min, and the mobile phase comprised acetonitrile and formic acid with a gradient ratio; the injection volume was 10 μL. During analyses, the ESI parameters were set as follows: capillary voltage, 3.6 kV for negative mode; desolvation temperature, 350 °C; cone gas flow, 100 L/h; and desolvation gas flow, 650 L/h.

PK parameters were represented as the mean and standard deviation (SD) from individual rats from each group and were estimated through noncompartmental analysis. The terminal elimination rate constant (K_e_) was estimated from the slope of the log–linear phase of a graph of the declining plasma concentration of curcumin versus time. The half-life (T_1/2_) was calculated using the following equation: T_1/2_ = ln 2/K_e_. Furthermore, the AUC from the beginning to the end point (AUC_0 → last_) was calculated using the trapezoidal method. Summing AUC_0→last_ and the concentration at the last measured point divided by K_e_ yielded AUC_0→∞_. Clearance (CL) was calculated by dividing the dose by AUC_0→∞_, and the distribution volume (V) was calculated by dividing CL by K_e_.

### Statistical analysis

Data are presented as the mean ± SD. The Student’s *t* test was used for assessing unequal variance. A 2-tailed *p* value less than 0.05 was considered significant.

## Additional Information

**How to cite this article**: Chen, L.-C. *et al*. Development and Characterization of Lecithin-based Self-assembling Mixed Polymeric Micellar (*sa*MPMs) Drug Delivery Systems for Curcumin. *Sci. Rep.* **6**, 37122; doi: 10.1038/srep37122 (2016).

**Publisher’s note**: Springer Nature remains neutral with regard to jurisdictional claims in published maps and institutional affiliations.

## Figures and Tables

**Table 1:** Preliminary screening of mixed micelle formation with various ratios of different amphiphiles (NaDOC; TPGS; PLURONIC P123, F68, F87, F127, F108, and L121; CREMOPHOR RH40 and ELP) and lecithin.

Amphiphiles (HLB)	Curcumin:Lecithin:Amphiphile					
	1:0:5	1:1:5	1:0:10	1:1:10	1:0:20	1:1:20
NaDOC (16)	104.4 ± 2.23^a^ (0.691 ± 0.045)^b^	91.1 ± 4.77 (0.564 ± 0.006)	109.9 ± 4.96 (0.498 ± 0.169)	78.8 ± 2.59 (0.294 ± 0.098)	111.6 ± 4.24 (0.327 ± 0.021)	132.7 ± 1.82 (0.308 ± 0.042)
TPGS (13)	145.9 ± 49.35 c (1.457 ± 0.311)	286.9 ± 21.26 (1.250 ± 0.153)	44.3 ± 27.11 (−6.385 ± 5.218)	49.6 ± 2.98 (1.140 ± 0.109)	43.8 ± 1.20 (0.708 ± 0.181)	47.2 ± 6.54 (1.583 ± 0.040)
F68 ( > 24)	131.5 ± 10.60 (0.230 ± 0.039)	403.1 ± 83.86 (0.943 ± 0.192)	83.1 ± 23.36 (0.107 ± 0.062)	270.1 ± 6.86 (0.967 ± 0.022)	139.6 ± 59.21 (1.221 ± 0.437)	338.2 ± 10.73 (0.756 ± 0.084)
F108 ( > 24)	279.8 ± 203.15 (1.728 ± 0.176)	800.0 ± 20.98 (1.134 ± 0.132)	126.6 ± 28.23 (1.310 ± 0.559)	430.7 ± 10.12 (1.314 ± 0.023)	279.8 ± 203.15 (1.728 ± 0.176)	316.9 ± 5.00 (1.061 ± 0.014)
F87 ( > 24)	67.0 ± 3.62 (0.303 ± 0.093)	410.8 ± 5.32 (0.627 ± 0.052)	67.5 ± 11.12 (1.579 ± 0.069)	488.3 ± 38.48 (0.888 ± 0.084)	47.3 ± 21.23 (1.435 ± 0.136)	596.0 ± 118.27 (1.042 ± 0.044)
F127 (18–23)	92.1 ± 74.83 (−0.293 ± 1.562)	546.4 ± 95.08 (1.183 ± 0.250)	36.6 ± 5.89 (−0.060 ± 0.730)	421.3 ± 16.91 (0.905 ± 0.104)	244.6 ± 40.43 (1.680 ± 0.180)	322.9 ± 4.20 (1.419 ± 0.069)
L121 (1–7)	>3000	>3000	>3000	>3000	>3000	>3000
P123 (7–9)	111.2 ± 1.52 (0.334 ± 0.043)	106.1 ± 0.84 (0.820 ± 0.035)	85.1 ± 4.11 (1.236 ± 0.157)	622.8 ± 68.14 (1.881 ± 0.161)	69.1 ± 3.04 (1.376 ± 0.085)	257.8 ± 43.38 (2.116 ± 0.062)
RH40 (14.3)	257.4 ± 57.46 (−0.060 ± 0.037)	264.1 ± 73.32 (0.860 ± 0.313)	71.5 ± 101.12 (0.021 ± 0.353)	742.1 ± 87.89 (−3.450 ± 1.025)	43.0 ± 18.65 (1.294 ± 0.414)	208.9 ± 11.63 (1.960 ± 0.071)
ELP (13.9)	290.9 ± 6.50 (0.598 ± 0.031)	429.8 ± 9.48 (1.617 ± 0.017)	42.6 ± 36.48 (0.527 ± 0.666)	534.3 ± 206.48 (1.392 ± 0.174)	25.4 ± 11.79 (1.619 ± 0.174)	216.0 ± 1.14 (0.372 ± 0.024)

^a^Mean size ± SD.

^b^Mean PI ± SD.

^c^Underline stander for precipitation during 12 hr.

**Table 2:** Particle size, polydispersity index (P.I.), drug loading (D.L.), and encapsulation efficiency (E.E.) of lecithin-based mixed curcumin micelles formed using different amphiphilic polymers (NaDOC, PLURONIC P123, and TPGS) at different ratios.

C:L:NaDOC	Particle size (nm)	P.I.	D.L. (%)	E.E. (%)
1:0:5	71.6 ± 11.28	0.491 ± 0.209	14.06	84.36
2:1:10	112.9 ± 1.09	0.458 ± 0.060	13.75	89.39
1:1:5	89.4 ± 4.21	0.551 ± 0.038	12.47	87.32
1:2:5	102.3 ± 6.15	0.331 ± 0.083	11.10	88.81
2:0:5	137.5 ± 0.71	0.222 ± 0.038	27.80	97.29
2:1:5	83.9 ± 5.02	1.059 ± 0.334	21.91	87.64
2:2:5	113.3 ± 1.54	0.236 ± 0.035	18.32	82.43
3:0:10	251.8 ± 5.91	0.907 ± 0.054	22.62	98.00
3:1:10	94.3 ± 4.32	0.497 ± 0.086	17.96	83.82
3:2:10	110.1 ± 0.58	0.155 ± 0.024	16.95	84.75
1:0:10	107.6 ± 7.01	0.631 ± 0.104	7.04	77.42
2:1:20	99.4 ± 0.25	0.297 ± 0.018	7.80	89.73
1:1:10	88.5 ± 3.05	0.325 ± 0.111	6.55	78.56
1:2:10	111 ± 2.04	0.115 ± 0.002	6.84	88.97
1:0:20	93.5 ± 2.31	0.283 ± 0.073	3.81	79.96
1:1:20	83 ± 1.45	0.323 ± 0.039	3.81	83.88
1:2:20	128.7 ± 5.4	0.410 ± 0.091	3.85	88.57
**C:L:P123**	**Particle size (nm)**	**P.I.**	**D.L. (%)**	**E.E. (%)**
3:1:10	139.5 ± 0.82	0.479 ± 0.034	17.70	82.60
3:2:10	152.8 ± 3.17	0.367 ± 0.067	17.27	86.35
5:0:20	116.9 ± 1.11	0.563 ± 0.038	14.25	74.10
5:2:20	137.9 ± 1.90	0.397 ± 0.096	16.41	88.64
1:0:5	115.6 ± 0.53	0.357 ± 0.019	13.60	82.78
4:1:20	77.3 ± 1.49	0.258 ± 0.093	13.20	82.53
1:1:5	97.5 ± 0.67	0.667 ± 0.106	12.09	84.65
3:1:20	71.3 ± 4.69	0.877 ± 0.316	8.53	68.21
3:2:20	91.1 ± 2.94	0.662 ± 0.157	10.25	85.44
1:0:10	59.6 ± 26.73	−2.328 ± 5.463	7.29	80.17
1:0:20	56.1 ± 20.35	1.924 ± 0.526	3.79	79.56
1:1:20	154 ± 16.29	1.836 ± 0.075	3.91	86.14
**C:L:TPGS**	**Particle size (nm)**	**P.I.**	**D.L. (%)**	**E.E. (%)**
1:0:5	121.4 ± 1.33	0.589 ± 0.076	11.62	69.72
1:1:5	97.9 ± 1.19	0.845 ± 0.102	11.86	83.05
1:2:5	126.1 ± 4.46	0.874 ± 0.165	10.50	83.99
1:0:10	22.7 ± 2.32	1.159 ± 0.493	6.93	76.20
1:1:10	42.6 ± 2.1	0.877 ± 0.174	6.15	73.82
1:2:10	85.8 ± 8.06	0.925 ± 0.445	6.30	81.95
1:0:20	29.2 ± 3.14	0.554 ± 0.582	3.35	70.29
1:1:20	39.9 ± 6.03	1.321 ± 0.216	3.96	87.13
5:2:20	106.1 ± 5.58	0.652 ± 0.081	13.44	72.58

**Table 3:** Summary of pharmacokinetic parameters of curcumin following iv administration of lecithin-based mixed micelles (NaDOC and PLURONIC P123) and free curcumin or oral administration of lecithin-based mixed micelles (NaDOC and PLURONIC P123) and free curcumin (n = 3).

Treatment group	IV_Free_	IV_P123_^a^	IV_NaDOC_^b^	Oral_Free_	Oral_P123_^a^	Oral_NaDOC_^b^
Dose (mg/kg)	5	5	5	100	100	100
k_el_ (1/hr)	0.17 ± 0.08	0.05 ± 0.04	0.03 ± 0.03	0.07	0.92 ± 1.44	0.25 ± 0.25
t_1/2_ (h)	4.43 ± 1.56	16.80 ± 8.08	26.90 ± 13.33	10.41	4.94 ± 4.07	14.05 ± 20.49
T_max_ (h)				1.75	0.33 ± 0.14	0.25 ± 0.00
C_max_ (μg/ml)				0.004 ± 0.003	0.07 ± 0.02	0.06 ± 0.05
AUC_0–72_ (h.μg/ml)	0.37 ± 0.15	0.86 ± 0.20	2.12 ± 1.53	0.03 ± 0.06	0.09 ± 0.017	0.15 ± 0.18
V (L/kg)	91.28 ± 43.1	138.82 ± 62.73	100.83 ± 21.03	14387.49	7104.32 ± 5701.98	9931.80 ± 5739.07
CL (L/h/kg)	14.69 ± 4.88	5.99 ± 1.35	3.19 ± 1.80	958.04	1109.8 ± 209.94	1549.76 ± 1154.73
F_ab_ (%)	100**	232**	573	0.4**	1.2**	2.0**
F_rel_ (%)				100	300	500

^a^Curcumin/Lecithin/PLURONIC P123 = 5/2/20;

^b^Curcumin/Lecithin/NaDOC = 6/2/5.

## References

[b1] Sharma R. A., Gescher A. J. & Steward W. P. Curcumin: The story so far. Eur. J. Cancer 41, 1955–1968 (2005). 16081279 10.1016/j.ejca.2005.05.009

[b2] Strimpakos A. S. & Sharma R. A. Curcumin: Preventive and therapeutic properties in laboratory studies and clinical trials. Antioxid. Redox. Signal. 10, 511–545(2008). 18370854 10.1089/ars.2007.1769

[b3] Wahlang B., Pawar Y. B. & Bansal A. K. Identification of permeability-related hurdles in oral delivery of curcumin using the Caco-2 cell model. Eur. J. Pharm. Biopharm. 77, 275–282 (2011). 21147222 10.1016/j.ejpb.2010.12.006

[b4] Chen A. L. . Phase I clinical trial of curcumin, a chemopreventive agent, in patients with high-risk or pre-malignant lesions. Anticancer Res. 21, 2895–2900 (2001). 11712783

[b5] Aggarwal B., Sundaram C., Malani N. & Ichikawa H. Curcumin: The Indian solid gold. In Advances in Experimental Medicine and Biology (Eds. Aggarwal B. B., Surh Y. J. & Shishodia S.) 1–75 (Springer, 2007). 10.1007/978-0-387-46401-5_1 17569205

[b6] Kaminaga Y. . Production of unnatural glucosides of curcumin with drastically enhanced water solubility by cell suspension cultures of Catharanthus roseus. FEBS Lett. 555, 311–316 (2003). 14644434 10.1016/s0014-5793(03)01265-1

[b7] Anand P., Kunnumakkara A. B., Newman R. A. & Aggarwal B. B. Bioavailability of curcumin: Problems and promises. Mol. Pharm. 4, 807–818 (2007). 17999464 10.1021/mp700113r

[b8] Pan M. H., Huang T. M. & Lin J. K. Biotransformation of curcumin through reduction and glucuronidation in mice. Drug Metab. Dispos. 27, 486–494 (1999). 10101144

[b9] Suresh D. & Srinivasan K. Studies on the *in vitro* absorption of spice principles - Curcumin, capsaicin and piperine in rat intestines. Food Chem. Toxicol. 45, 1437–1442 (2007). 17524539 10.1016/j.fct.2007.02.002

[b10] Takahashi M., Uechi S., Takara K., Asikin Y. & Wada K. Evaluation of an oral carrier system in rats: Bioavailability and antioxidant properties of liposome-encapsulated curcumin. J. Agric. Food Chem. 57, 9141–9146 (2009). 19757811 10.1021/jf9013923

[b11] Maiti K., Mukherjee K., Gantait A., Saha B. P. & Mukherjee P. K. Curcumin-phospholipid complex: Preparation, therapeutic evaluation and pharmacokinetic study in rats. Int. J. Pharm. 330, 155–163 (2007). 17112692 10.1016/j.ijpharm.2006.09.025

[b12] Bisht S., Feldmann G., Soni S., Ravi R., Karikar C. & Maitra A. Polymeric nanoparticle-encapsulated curcumin (“nanocurcumin”): A novel strategy for human cancer therapy. J. Nanobiotechnology 5, 3 p1-18 (2007). 10.1186/1477-3155-5-3 PMC1868037 17439648

[b13] Anand P. . Design of curcumin-loaded PLGA nanoparticles formulation with enhanced cellular uptake, and increased bioactivity *in vitro* and superior bioavailability *in vivo*. Biochem. Pharmacol. 79, 330–338 (2010). 19735646 10.1016/j.bcp.2009.09.003 PMC3181156

[b14] Shaikh J., Ankola D. D., Beniwal V., Singh D. & Kumar M. N. V. R. Nanoparticle encapsulation improves oral bioavailability of curcumin by at least 9-fold when compared to curcumin administered with piperine as absorption enhancer. Eur. J. Pharm. Sci. 37, 223–230 (2009). 19491009 10.1016/j.ejps.2009.02.019

[b15] Mohanty C. & Sahoo S. K. The *in vitro* stability and *in vivo* pharmacokinetics of curcumin prepared as an aqueous nanoparticulate formulation. Biomaterials 31, 6597–6611 (2010). 20553984 10.1016/j.biomaterials.2010.04.062

[b16] Gou M. . Curcumin-loaded biodegradable polymeric micelles for colon cancer therapy *in vitro* and *in vivo*. Nanoscale 3, 1558–1567 (2011). 21283869 10.1039/c0nr00758g

[b17] Gong J., Chen M., Zheng Y., Wang S. & Wang Y. Polymeric micelles drug delivery system in oncology. J. Control. Release 159, 312–323 (2012). 22285551 10.1016/j.jconrel.2011.12.012

[b18] Lu Y. & Park K. Polymeric micelles and alternative nanonized delivery vehicles for poorly soluble drugs. Int. J. Pharm. 453, 198–214 (2013). 22944304 10.1016/j.ijpharm.2012.08.042 PMC3760723

[b19] Jones M. & Leroux J. Polymeric micelles - a new generation of colloidal drug carriers. Eur. J. Pharm. Biopharm. 48, 101–111 (1999). 10469928 10.1016/s0939-6411(99)00039-9

[b20] Yokoyama M. Polymeric micelles as a new drug carrier system and their required considerations for clinical trials. Expert Opin. Drug Del. 7, 145–158 (2010). 10.1517/17425240903436479 20095939

[b21] Maeda H., Wu J., Sawa T., Matsumura Y. & Hori K. Tumor vascular permeability and the EPR effect in macromolecular therapeutics: A review. J. Control. Release 65, 271–284 (2000). 10699287 10.1016/s0168-3659(99)00248-5

[b22] Maeda H., Sawa T. & Konno T. Mechanism of tumor-targeted delivery of macromolecular drugs, including the EPR effect in solid tumor and clinical overview of the prototype polymeric drug SMANCS. J. Control. Release 74, 47–61 (2001). 11489482 10.1016/s0168-3659(01)00309-1

[b23] Wei Z. . Paclitaxel-loaded PLURONIC P123/F127 mixed polymeric micelles: formulation, optimization and *in vitro* characterization. Int. J. Pharm. 376, 176–185 (2009). 19409463 10.1016/j.ijpharm.2009.04.030

[b24] Krishnadas A., Rubinstein I. & Onyuksel H. Sterically stabilized phospholipid mixed micelles: *in vitro* evaluation as a novel carrier for water-insoluble drugs. Pharm. Res. 20, 297–302 (2003). 12636171 10.1023/a:1022243709003

[b25] Jin X. . A novel drug-phospholipid complex loaded micelle for baohuoside I enhanced oral absorption: *in vivo* and *in vivo* evaluations. Drug Dev. Ind. Pharm. 39, 1421–1430 (2013). 23057574 10.3109/03639045.2012.719234

[b26] Marczylo T. H. . Comparison of systemic availability of curcumin with that of curcumin formulated with phosphatidylcholine. Cancer Chemother. Pharmacol. 60, 171–177 (2007). 17051370 10.1007/s00280-006-0355-x

[b27] Yanasarn N., Sloat B. R. & Cui Z. Nanoparticles engineered from lecithin-in-water emulsions as a potential delivery system for docetaxel. Int. J. Pharm. 379, 174–180 (2009). 19524029 10.1016/j.ijpharm.2009.06.004 PMC2736872

[b28] Hu K., Cao S., Hu F. & Feng J. Enhanced oral bioavailability of docetaxel by lecithin nanoparticles: Preparation, *in vitro*, and *in vivo* evaluation. Int. J. Nanomed. 7, 3537–3545 (2012). 10.2147/IJN.S32880 PMC3405895 22848177

[b29] Yanyu X., Yunmei S., Zhipeng C. & Qineng P. The preparation of silybin-phospholipid complex and the study on its pharmacokinetics in rats. Int. J. Pharm. 307, 77–82 (2006). 16300915 10.1016/j.ijpharm.2005.10.001

[b30] Cuomo J. . Comparative absorption of a standardized curcuminoid mixture and its lecithin formulation. J. Nat. Prod. 74, 664–669 (2011). 21413691 10.1021/np1007262

[b31] Chen L. C. . Development and characterization of self-assembling lecithin-based mixed polymeric micelles containing quercetin in cancer treatment and an *in vivo* pharmacokinetic study. Int. J. Nanomed. 11, 1557–1566 (2016). 10.2147/IJN.S103681 PMC4841422 27143878

[b32] Zweers M. L. T., Grijpma D. W., Engbers G. H. M. & Feijen J. The Preparation of Monodisperse Biodegradable Polyester Nanoparticles with a Controlled Size. J. Biomed. Mater. Res. Part B Appl. Biomater. 66, 559–566 (2003). 12861608 10.1002/jbm.b.10046

[b33] Yen F. L., Wu T. H., Lin L. T., Cham T. M. & Lin C. C. Nanoparticles formulation of Cuscuta chinensis prevents acetaminophen-induced hepatotoxicity in rats. Food Chem. Toxicol. 46, 1771–1777 (2008). 18308443 10.1016/j.fct.2008.01.021

[b34] Vareed S. K. . Pharmacokinetics of curcumin conjugate metabolites in healthy human subjects. Cancer Epidemiol. Biomarkers Prev. 17, 1411–1417 (2008). 18559556 10.1158/1055-9965.EPI-07-2693 PMC4138955

[b35] Tønnesen H. H., Másson M. & Loftsson T. Studies of curcumin and curcuminoids. XXVII. Cyclodextrin complexation: Solubility, chemical and photochemical stability. Int. J. Pharm. 244, 127–135 (2002). 12204572 10.1016/s0378-5173(02)00323-x

[b36] Tønnesen H. H. Solubility and stability of curcumin in solutions containing alginate and other viscosity modifying macromolecules: Studies of curcumin and curcuminoids XXX. Pharmazie 61, 696–700 (2006). 16964713

[b37] Alexandridis P. & Hatton T. Alan. Poly(ethylene oxide)poly(propylene oxide)poly(ethylene oxide) block copolymer surfactants in aqueous solutions and at interfaces: thermodynamics, structure, dynamics, and modeling, *Colloids Surf. A: Physicochem*. Eng. Asp. 96, 1–46 (1995).

[b38] Abdelbary G. A. & Tadros M. I. Brain targeting of olanzapine via intranasal delivery of core-shell difunctional block copolymer mixed nanomicellar carriers: *In vitro* characterization, *ex vivo* estimation of nasal toxicity and *in vivo* biodistribution studies. Int. J. Pharm. 452, 300–310 (2013). 23684658 10.1016/j.ijpharm.2013.04.084

[b39] Pepić I., Lovrić J., Hafner A. & Filipović-Grčić J. Powder form and stability of PLURONIC mixed micelle dispersions for drug delivery applications. Drug Dev. Ind. Pharm. 40, 944–951 (2014). 23627442 10.3109/03639045.2013.791831

[b40] Zhao Y., Li Y., Ge J., Li N. & Li L. B. PLURONIC-poly (acrylic acid)-cysteine/PLURONIC L121 mixed micelles improve the oral bioavailability of paclitaxel. Drug Dev. Ind. Pharm. 40, 1483–1493 (2014). 23971495 10.3109/03639045.2013.829487

[b41] Lee E. S. . Binary mixing of micelles using PLURONICs for a nano-sized drug delivery system. Colloids Surf. B Biointerfaces 82, 190–195 (2011). 20850281 10.1016/j.colsurfb.2010.08.033

[b42] Wang Y. J. . Stability of curcumin in buffer solutions and characterization of its degradation products. J. Pharm. Biomed. Anal. 15, 1867–1876 (1997). 9278892 10.1016/s0731-7085(96)02024-9

[b43] Ansari M. J., Ahmad S., Kohli K., Ali J. & Khar R. K. Stability-indicating HPTLC determination of curcumin in bulk drug and pharmaceutical formulations. J. Pharm. Biomed. Anal. 39, 132–138 (2005). 15941643 10.1016/j.jpba.2005.03.021

[b44] Tsai Y. M., Chien C. F., Lin L. C. & Tsai T. H. Curcumin and its nano-formulation: The kinetics of tissue distribution and blood-brain barrier penetration. Int. J. Pharm. 416, 331–338 (2011). 21729743 10.1016/j.ijpharm.2011.06.030

[b45] Xie X. . PLGA nanoparticles improve the oral bioavailability of curcumin in rats: Characterizations and mechanisms. J. Agric. Food Chem. 59, 9280–9289 (2011). 21797282 10.1021/jf202135j

[b46] Gao Y., Li L. B. & Zhai G. Preparation and characterization of PLURONIC/TPGS mixed micelles for solubilization of camptothecin. Colloids Surf. B Biointerfaces 64, 194–199 (2008). 18325744 10.1016/j.colsurfb.2008.01.021

[b47] Yang K. Y., Lin L. C., Tseng T. Y., Wang S. C. & Tsai T. H. Oral bioavailability of curcumin in rat and the herbal analysis from Curcuma longa by LC-MS/MS. J. Chromatogr. B: Anal. Technol. Biomed. Life Sci. 853, 183–189 (2007). 10.1016/j.jchromb.2007.03.010 17400527

[b48] Zhao L. . Formulation and *in vitro* evaluation of quercetin loaded polymeric micelles composed of PLURONIC P123 and D-α-tocopheryl polyethylene glycol succinate. J. Biomed. Nanotechnol. 7, 358–365 (2011). 21830476 10.1166/jbn.2011.1298

