# Corrigendum: Development and Characterization of Lecithin-based Self-assembling Mixed Polymeric Micellar (*sa*MPMs) Drug Delivery Systems for Curcumin

**DOI:** 10.1038/srep44967

**Published:** 2017-03-22

**Authors:** Ling-Chun Chen, Yin-Chen Chen, Chia-Yu Su, Wan-Ping Wong, Ming-Thau Sheu, Hsiu-O Ho

Scientific Reports 6: Article number: 37122; 10.1038/srep37122 published online: 11 16 2016; updated: 03 22 2017.

The authors neglected to cite a previous study related to the giving of a single oral dose of 410 mg micellar curcumin to women and men. This additional reference is listed below as reference 1, and should appear in the text as below.

In the Introduction section,

“Furthermore, the maximum concentration (Cmax) achieved using a single oral dose of 410 mg of curcumin micelles (women, 3.7 μmol/L; men 2.6 μmol/L) was higher than that achieved using 8 g of free curcumin^4^”.

should read:

“Furthermore, the maximum concentration (Cmax) achieved using a single oral dose of 410 mg of curcumin micelles (women, 3.7 μmol/L; men 2.6 μmol/L)^1^ was higher than that achieved using 8 g of free curcumin^4^”.
